# A mixed-methods approach utilising electronic health records to examine antimicrobial prescription surrounding gastrointestinal clinical presentations in dogs and cats

**DOI:** 10.3389/fvets.2023.1166114

**Published:** 2023-12-12

**Authors:** Ivo S. Fins, David A. Singleton, Alan D. Radford, Fernando Sánchez-Vizcaíno, Gina L. Pinchbeck

**Affiliations:** ^1^Institute of Infection, Veterinary and Ecological Sciences, Department of Livestock and One Health, University of Liverpool, Neston, United Kingdom; ^2^Institute of Infection, Veterinary and Ecological Sciences, Department of Infection Biology and Microbiomes, University of Liverpool, Neston, United Kingdom; ^3^Bristol Veterinary School, University of Bristol, Bristol, United Kingdom

**Keywords:** antimicrobial prescription, electronic health records, gastrointestinal presentations, companion animals, veterinary health informatics, mixed-methods

## Abstract

**Introduction:**

Systemically-administered antimicrobials are often prescribed in canine and feline gastrointestinal clinical presentations. Responsible use of antimicrobials, particularly those considered Highest Priority Critically Important Antimicrobials (HPCIAs) is vital to tackle antimicrobial resistance. Although practice-level prescription guidance is available, further strategies based on a greater understanding of antimicrobial prescription at the population-level are needed. Here, we used a mixed-methods approach, harnessing veterinary electronic health records (EHRs) to characterise the use of antimicrobials in canine and feline gastrointestinal presentations, and to explore justification and reasoning around antimicrobial prescribing, particularly of HPCIAs.

**Methods:**

This observational study used 23,337 EHRs complemented with veterinary practitioner-completed questionnaires, from canine and feline gastrointestinal consultations from 225 volunteer UK veterinary practices between April 2014 and September 2018.

**Results:**

A total of 83.4% (95% confidence interval (CI) 82.6–84.3) gastrointestinal presentations were reported as mild, with non-haemorrhagic diarrhoea and vomiting the most frequently reported clinical signs. Systemically-administered antimicrobials occurred in 28.6% of canine (95% CI 26.9–30.3) and 22.4% of feline (95% CI 20.4–24.4) gastrointestinal consultations, with HPCIA prescription occurring more frequently in cats. Results of multivariable analysis showed the presence of non-haemorrhagic diarrhoea (canine Odds Ratio (OR) 2.1, 95% CI 1.9–2.3; feline OR 1.8, 95% CI 1.5–2.1), haemorrhagic diarrhoea (canine OR 4.2, 95% CI 3.8–4.7; feline OR 3.1, 95% CI 2.4–3.8), and moderate/severe presentations (canine OR 1.9, 95% CI 1.7–2.8; feline OR 2.0, 95% CI 1.7–2.5) were positively associated with receiving a systemically-administered antimicrobial. Thematic analysis of clinical narrative content of 516 gastrointestinal consultations where HPCIAs were prescribed allowed the identification of ten factors underpinning reasoning or decision-making for HPCIA prescription: perceived animal/owner compliance; owner’s expectations; perceived risk of infection; clinical signs; recent clinical history; perceived positive previous response to antimicrobial therapy; geriatric patients and euthanasia; concomitant conditions; diagnostic testing and the behavioral trend to trial antimicrobial therapy empirically in gastrointestinal cases. No explicit justification for HPCIA prescription was recorded in 77% of cases.

**Discussion:**

Improving recorded justification represents a clear target for stewardship programmes. By utilising a complementary mixed-methods approach to EHRs, this study unlocks previously untapped data recorded within EHRs. These results can help inform targeted interventions, contributing towards enhanced antimicrobial stewardship.

## 1 Introduction

Antimicrobial resistance (AMR) is a complex global health problem involving different bacterial species, resistance mechanisms, and reservoirs. Selection pressure associated with antimicrobial use is one of the most important factors responsible for increased AMR (1, 2). Evidence of development of resistance in response to treatment and transmission of bacterial resistance amongst human beings and companion animals demonstrates the necessity of an interdisciplinary approach to preserve antimicrobial efficacy, which requires identification of opportunities to safely reduce antimicrobial prescriptions (AMPs) (3–7).

Gastrointestinal (GI) disease is a common reason for seeking veterinary care for small animals in the United Kingdom (8), with a multifactorial aetiology spanning from self-limiting factors (e.g., dietary indiscretion) to potentially life-threatening causes (e.g., canine and feline parvoviruses) (8–10). Bacteria have been implicated in canine and feline diarrhoea, including zoonotic pathogens such as *Clostridium*, *Salmonella*, and *Campylobacter* spp. (11). Nonetheless, the exact role of these enteropathogens remains under debate (11, 12). Dogs and cats presenting with GI signs are often treated symptomatically in the absence of specific diagnostic testing (9, 13, 14). This empirical approach often includes antimicrobial prescription (AMP) as a treatment strategy (3, 14). Efforts to encourage responsible veterinary antimicrobial use have been developed, such as the recently published *‘categorisation of antibiotics for use in animals for prudent and responsible use,’* by the European Medicines Agency (EMA) (15), as well as practise-level guidance, such as ‘PROTECT ME’ (16). However, there is a need to understand how these policies are reflected in practise and what key factors may influence AMP in canine and feline GI clinical presentations.

Different studies using quantitative methodologies and electronic health records (EHRs) have helped establish the typical profile of gastrointestinal clinical presentations, including a diagnostic approach and therapeutic and other management choices (3, 8). Following their application in human health (17–19), complementary qualitative approaches, such as using in-depth interviews, have also been used to better understand veterinary antimicrobial prescribing behaviour in both food and companion animals (20–23).

A greater understanding of AMP at a population level is needed for the veterinary profession, especially for highest priority critically important antimicrobials (HPCIAs) as defined by the World Health Organisation (24), such as fluoroquinolones, macrolides, and third-generation cephalosporins. Whilst quantitative studies can describe and quantify antimicrobial use, they are not able to describe the key drivers of AMP during consultations. The aims of this study were therefore two-fold: (i) to characterise canine and feline GI clinical presentations and to explore risk factors associated with the prescription of systemically administered antimicrobials; (ii) in a subset of these cases, to describe the justification and/or reasoning around AMP, particularly associated with HPCIA prescription, as recorded at the time of an AMP event in the EHR.

## 2 Materials and methods

### 2.1 Data collection

This retrospective observational study analysed EHRs collected from a sentinel network of 225 United Kingdom volunteer veterinary practises (502 sites) participating in the small animal veterinary surveillance network (SAVSNET) and operating Robovet practise management software (Vet Solutions Ltd.). A “veterinary practise” was defined as a single business, whilst “veterinary site” included all branches that form a practise. Data collection protocols are more extensively described elsewhere (14, 25). In this study, EHRs were collected from consultations where an appointment was made to see a veterinary professional (veterinary surgeon or nurse) between 1 April 2014 and 30 September 2018. Each consultation is also mandatorily classified by the attending veterinary professional according to the main reason that the animal presented by choosing 1 of 10 main presenting complaints (MPCs), one of which is GI disease (3). Additionally, a short questionnaire (Table 1) was completed by the attending veterinary professional in a random selection of approximately 10% of consultations. This strategy aims to overcome issues related to the variable recording of details within the clinical narrative of EHRs, as previously described, but without introducing bias (14). All consultations that had been classified using the GI MPC which also had a completed questionnaire were selected for inclusion in this study.

**Table 1:** Questions provided to attending veterinary professionals (in approximately 10% of consultations, selected at random) where veterinary professionals had selected “gastrointestinal” as the main presenting complaint.

Question	Answer options
1. Please indicate the clinical signs present	Diarrhoea without blood; Diarrhoea with blood; Vomiting without blood; Vomiting with blood; Melaena; Weight loss/failure to gain weight; Poor appetite; Other
2. If diarrhoea was present, how would you describe it?	No diarrhoea; Small intestinal diarrhoea; Large intestinal diarrhoea/colitis; Mixed pattern; Do not know
3. Please indicate disease severity	Mild illness, i.e., normal apart from GI disease; Moderately ill; Severely ill/debilitated
4. How does this consultation relate to this episode of illness?	First presentation; Revisit/check-up; Do not know
5. How long approximately has the pet had this episode of illness?	Up to 2 days; Between 3 days and 2 weeks; More than 2 weeks and less than 1 month; 1 month and over; Do not know
6. What diagnostic options will be used today for this episode of illness?	None; Faecal parasitology/bacteriology; Faecal virology; Virus serology; Diagnostic Imaging; Haematology/biochemistry; Serum; B12/Folate and/or serum TLI; Canine/feline specific pancreatic lipase; Urinalysis; Other
7. What advice did you give today?	Change of diet; Fasting; Admit patient for treatment; Refer patient; Check-up in near future; Other

Consultations where diarrhoea and/or vomiting were indicated as present on the questionnaire were included. Only consultations where the episode of the veterinary visit was clearly defined by the attending veterinary professional on the questionnaire were included in the study (i.e., one option selected to define the visit episode as *‘first visit’* or *‘revisit’*). Consultations were excluded if multiple answers on visit episode and/or *‘do not know’* were selected on the questionnaires. In addition to the MPC and the associated questionnaire responses, each EHR also included signalment data, such as age, sex, neutered status, a text-based product dispensed description, and any vaccination history. Animals were defined as vaccinated if they had a recorded vaccination of any composition within 3.5 years prior to the consultation date (14, 26).

Pharmaceutical product prescription of the most common GI-active agents was described using five pharmaceutical families (14, 27), namely, antimicrobial agents authorised for systemic use (injectable or oral formulations, hence “systemically administered”), anti-inflammatory drugs authorised for systemic use, antiparasitic agents (endoparasiticides or endectocides), gastrointestinally active products, such as proton pump inhibitors, and products used for euthanasia (hereafter “euthanasia”). Gastrointestinal nutraceuticals were also included in the dispensed product analyses. These were defined as products not listed as either authorised veterinary or human medicinal products, which contained a range of probiotics, prebiotics, kaolin, etc., and were likely dispensed with the purpose of assisting diarrhoea resolution (14).

### 2.2 Statistical analyses

Statistical analyses were carried out using R (version 3.5.0). Descriptive proportions and associated 95% confidence intervals (95% CI) were calculated to adjust for clustering (bootstrap method, n = 5,000 samples) within a veterinary site, including a range of binary or categorical signalment, clinical sign, pharmaceutical agent prescription, and professional advice variables. Median and range were calculated for continuous variables. Univariable and multivariable mixed effects logistic regression models were fitted separately for dogs and cats using the R package ‘lme4’ to model on a GI case level the outcome variable ‘presence of systemically administered AMP’ against eight categorical covariates and one continuous variable (age). Likelihood ratio tests (LRT), Akaike Information Criterion (AIC), and Bayesian Information Criterion (BIC) were used to examine the presence of clustering within veterinary practise or site and were subsequently included in each constructed model as random effects according to whether each individually or both combined provided the best fit.

Initial univariable mixed effects logistic regression considered categorical factors related to animal signalment (insurance status, vaccination status, and neutered status) or questionnaire responses (consultation episode; faecal bacteriology/parasitology diagnostic testing; presence of diarrhoea and vomiting, including haemorrhagic and non-haemorrhagic; duration of the illness; and case severity). Considering case severity, due to a low number of severe cases, such cases were merged with moderate cases into a single category. For the continuous variable age, cubic polynomial terms were included if an LRT, AIC, and BIC indicated a significantly improved fit compared to linear and lesser polynomial terms. The projected AMP probability and associated 95% confidence intervals were calculated from log odds using ‘sjPlot’ (28). Explanatory variables were retained for multivariable analysis if an LRT indicated a *p*-value of ≤0.20 against a null model. Multivariable models underwent manual step-wise backward elimination to minimise AIC and BIC. Confounding was accounted for via the assessment of effect variation upon removal of variables. Two-way interaction terms between explanatory variables were assessed via AIC, BIC, and an LRT. The variance inflation factor (VIF) was used to assess multicollinearity. Statistical significance was defined as a *p*-value of <0.05.

### 2.3 Thematic analysis

All 516 anonymised clinical narratives where an HPCIA agent was prescribed were transferred into NVivo 12 (QSR) software for data management. A thematic analysis was utilised (29). Although thematic analysis is widely used in qualitative research, it is poorly defined as a methodology, with approaches taken being diverse and occasionally variable (30–32). Hence, to ensure consistency of data analysis, the six-phase approach to thematic analysis as defined by Braun and Clarke was adopted (20, 22, 31, 33). Thematic analysis can be defined as a method that allows to identify, analyse, and report patterns (defined as themes) within data whilst organising and describing the dataset in detail, thereby allowing the interpretation of different aspects related to the research topic (31). During the coding process, the whole clinical narrative was read and data extracts related to AMP were coded using a theoretical approach to the research question, which was underpinned by previously published qualitative studies regarding drivers of antimicrobial prescription and AMR in the small animal veterinary sector (22, 23, 34, 35). Rigour in the coding was ensured by a second researcher independently reading and classifying the narratives associated with the HPCIA prescribing EHRs (n = 503 out of 516, 13 EHRs pre-excluded from the review process as they were blank clinical narratives). Divergence in coding, interpretation, and themes was discussed and adjusted accordingly (36), and only minor discrepancy was found from the review process in 2 out of the 10 themes identified, which was related to the nomenclature of the themes and capturing the underlying behaviour (e.g., perceived efficacy vs. perceived positive previous response to antimicrobial therapy). Subsequently, the themes were refined to ensure that each was meaningful and clear but distinct from other themes (37). Minor themes that were linked by a common topic area, or that related to an overall topic, were grouped together and considered as major themes. A thematic map was constructed to review the relationships between minor and major themes. Quotes extracted from clinical narratives presented here as examples were selected to illustrate themes and are presented with the species of the animal given between square brackets. Where a typographical error, abbreviation, or acronym was present in the quote, the correct spelling and/or full word is given in square brackets after the misspelled/abbreviated word or acronym. A list of abbreviations and clinical acronyms found throughout the clinical quotes is also presented in Supplementary material, Section 2.

## 3 Results

### 3.1 Study population

**Figure 1:**
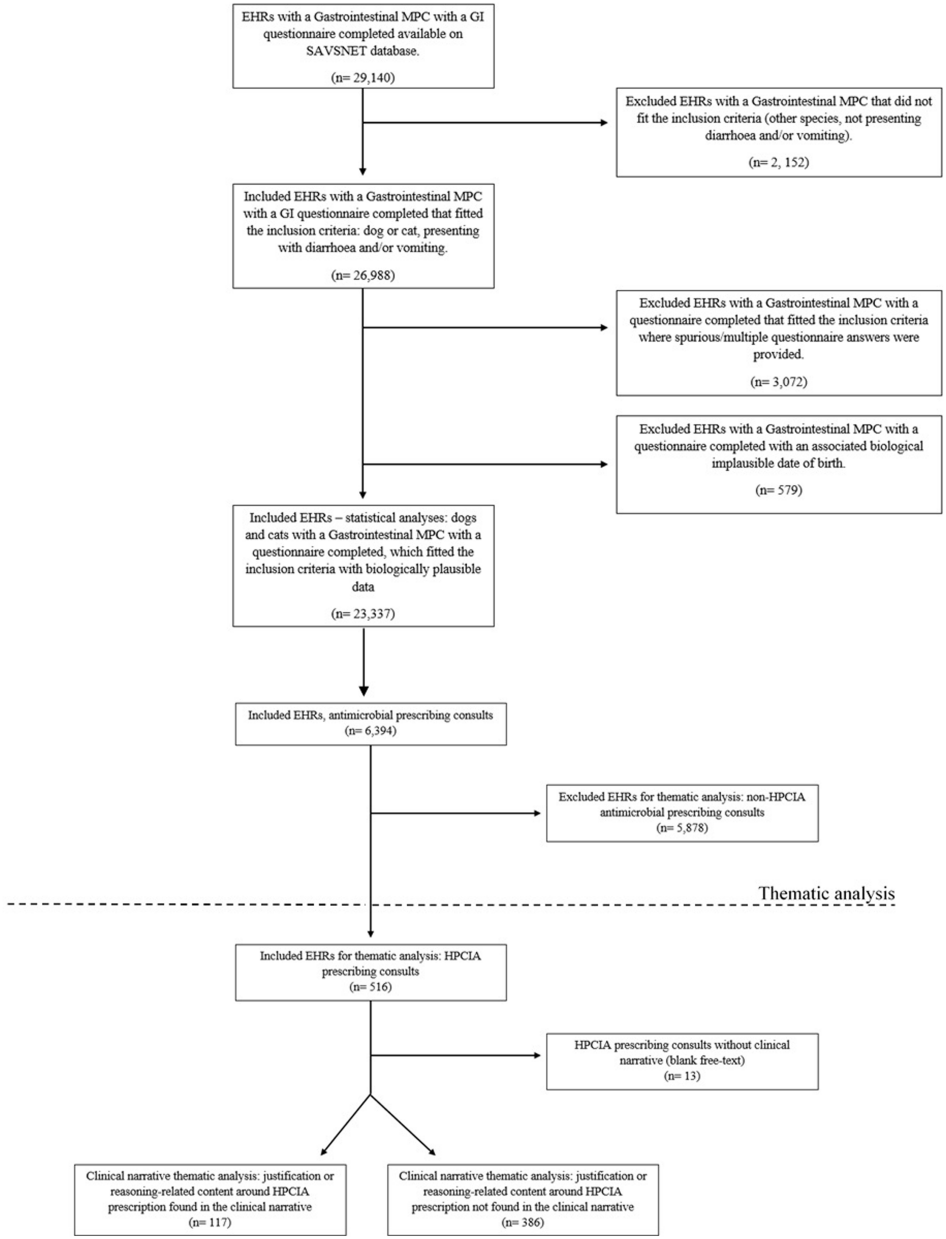
Study flow diagram.

A total of 29,140 EHRs were associated with a GI MPC and had a questionnaire completed, of which 26,988 fitted the inclusion criteria (dog or cat, presenting with diarrhoea and/or vomiting). EHRs containing a likely incorrect date of birth were removed (*n* = 579) (age range included for dogs: 0 to 29 years old; age range considered for cats: 0 to 38 years old), as were EHRs where spurious/multiple questionnaire answers were provided (*n* = 3,072). Consequently, 23,337 GI consultations collected from 225 volunteer veterinary practises (502 sites) were included in the initial descriptive analyses. Of these, 18,829 EHRs (81%) were from dogs and 4,508 (19%) were from cats. The overall data flow is shown in Figure 1.

### 3.2 Characterising canine and feline GI clinical presentations

The study population was further characterised (Table 2). The majority of both canine and feline GI consultations were recorded as first-visit consultations (74 and 66%, respectively), with 83% of canine consultations and 82% of feline consultations recorded as mild presentations. Severe cases were merged with moderate cases into a single category (moderate/severe) due to a low number of severe cases (*n* = 247 in canine consultations and *n* = 77 in feline consultations). The most frequently reported clinical signs were non-haemorrhagic diarrhoea and non-haemorrhagic vomiting, followed by haemorrhagic diarrhoea in both canine and feline GI consultations. For feline GI presentations, the most frequent durations being reported were less than 2 days duration (40%). Regarding canine GI presentations, the majority (54%) were of less than 2 days duration. Diagnostic tests were uncommonly used, with faecal bacteriology and parasitology testing being performed in less than 8% of both canine and feline cases. Dietary modification was the most commonly provided advice to dog and cat owners (Table 3).

**Table 2:** Characterisation of the study population (*n* = 23,337) and descriptive summary of the characteristics of gastrointestinal (GI) clinical presentations and diagnostic options according to questionnaire responses and electronic health records (EHRs).

	Canine GI consultations	Feline GI consultations
	(*n* = 18,829 EHRs)	(*n* = 4,508 EHRs)
	% (95% CI)^a^	% (95% CI)
Male	51.8 (51.0–52.6)	50.5 (49.0–52.0)
Female	48.2 (47.3–49.1)	49.5 (48.0–51.1)
Insured	26.7 (24.9–28.5)	18.0 (16.1–19.8)
Neutered	68.1 (67.0–69.3)	82.0 (80.5–83.4)
Microchipped	55.7 (54.1–57.3)	39.8 (37.8–41.8)
Vaccinated^b^	75.2 (73.9–76.6)	59.2 (57.3–61.1)
Median age	5.1 years(range: 0.0–19.4)	7.8 years(range 0.0–23.0)
Episode		
First visit	74.1 (73.0–75.1)	66.1 (64.3–67.9)
Revisit	25.9 (24.8–27.0)	33.9 (32.1–35.7)
Disease severity		
Mild	83.4 (82.6–84.3)	81.6 (80.3–82.9)
Moderate/Severe	16.6 (15.7–17.4)	18.4 (17.1–19.7)
Clinical signs		
Non-haemorrhagic diarrhoea	47.9 (47.1–48.7)	44.9 (43.3–46.6)
Haemorrhagic diarrhoea	28.8 (28.1–29.5)	14.9 (13.9–16,0)
Non-haemorrhagic vomiting	43.0 (42.1–43.9)	49.4 (47.7–51.1)
Haemorrhagic vomiting	3.6 (3.3–3.8)	3.9 (3.3–4.5)
Poor appetite	13.3 (12.4–14.1)	11.8 (10.6–13.1)
Weight loss/fail to gain weight	3.4 (3.0–3.8)	10.0 (8.8–11.2)
Melaena	0.6 (0.5–0.8)	0.1 (0–0.3)
Other	1.8 (1.4–2.1)	1.9 (1.5–2.4)
Duration		
≤ 2 days	53.7 (52.7–54.8)	40.1 (38.5–41.8)
≥ 3 days and < 2 weeks	35.6 (34.7–36.5)	32.9 (31.5–34.3)
≥ 2 weeks and < 1 month	3.9 (3.6–4.2)	7.3 (6.1–8.1)
≥ 1 month	6.2 (5.8–6.7)	18.8 (17.2–20.3)
Do not know	0.5 (0.4–0.6)	0.8 (0.6–1.1)
Diagnostic option		
Faecal bacteriology/parasitology	7.9 (7.1–8.7)	7.8 (6.8–8.7)
Faecal virology	0.2 (0.2–0.3)	0.2 (0.1–0.4)
Virus serology	0.1 (0.0–0.1)	0.3 (0.1–0.4)
Diagnostic Imaging	2.2 (2.0–2.5)	2.5 (2.1–3.0)
Haematology/biochemistry	7.2 (6.7–7.7)	12.1 (10.8–13.3)
PLI (specific pancreatic lipase)	2.1 (1.8–2.4)	2.8 (2.2–3.5)
Serum B12 and/or TLI	1.8 (1.6–2.1)	2.7 (2.1–3.3)
Urinalysis	0.6 (0.5–0.7)	1.3 (0.9–1.6)
Other	4.3 (3.8–4.8)	5.8 (4.8–6.7)

^a^Percentage of EHRs (95% confidence interval). ^b^Vaccinated within the preceding 3.5 years.

**Table 3:** Descriptive summary of advice given (according to questionnaire responses), pharmaceutical prescriptions, and dispensing of nutraceutical products distributed by species.

	Dogs (*n* = 18,829 EHRs)	Cats (*n* = 4,508 EHRs)
	% (95% CI)^a^	% (95% CI)
Advice		
Diet change Check-up Fast Admit Refer	62.3 (60.5–64.0) 25.3 (23.8–26.9) 12.4 (11.0–13.8) 3.1 (2.7–3.6) 0.3 (0.2–0.4)	53.5 (51.3–55.7) 29.4 (27.4–31.3) 5.3 (4.5–6.2) 3.1 (2.4–3.7) 0.5 (0.3–0.7)
Other	50.1 (48.2–52.1)	51.0 (48.9–53.1)
Therapy		
Gastrointestinally active pharmaceuticals	40.4 (39.2–41.6)	33.8 (31.8–35.7)
Systemically administered antimicrobial Systemically administered HPCIA^b^ Systemically administered anti-inflammatory Endoparasiticide and/or endectocide	28.6 (26.9–30.3) 0.9 (0.4–1.3) 9.8 (7.9–11.6) 18.8 (17.2–20.4)	22.4 (20.4–24.4) 5.0 (4.1–5.9) 16.8 (14.7–19.0) 19.1 (17.5–20.6)
Gastrointestinal nutraceutical	41.7 (39.9–43.5)	23.1 (21.5–24.7)
Euthanasia/death	0.2 (0.1–0.2)	0.3 (0.2–0.5)

^a^Percentage of EHRs (95% confidence interval). NB: Some consults had more than one active pharmaceutical prescribed. ^b^HPCIA: Highest priority critically important antimicrobials, according to the WHO.

### 3.3 Pharmaceutical prescriptions and dispensing of nutraceutical products

Gastrointestinally active pharmaceutical products were prescribed in 40.4 and 33.8% of canine and feline GI consultations, respectively, including systemically administered anti-inflammatories and endoparasiticides/endectocides (Table 3). Gastrointestinal nutraceuticals were more commonly dispensed in canine (42%) than feline (23%) GI consultations. Systemic antimicrobials were prescribed in 29% of canine and 22% of feline GI consultations. Systemic HPCIA prescription was low in canine presentations (0.9%), whereas in feline consultations, systemic HPCIA prescription occurred in 5% of GI consultations (Table 3). Considering the prescribing consultations (dogs *n* = 5,384: cats = 1,010), clavulanic acid-potentiated amoxicillin was the most commonly prescribed systemically administered antimicrobial in feline consultations (37%) and accounted for 33% of prescriptions in canine consultations (Table 4). In canine consultations, metronidazole was the most commonly prescribed systemic antimicrobial (34%). A third-generation cephalosporin, cefovecin, was commonly prescribed in feline consultations (19%), contrasting with a very low prescription percentage (0.5%) in canine consultations. The relative percentages of systemically administered antimicrobials in prescribing consultations distributed by species and grouped according to the EMA *“categorisation of antibiotics for use in animals for prudent and responsible use”* (15) are presented in Table 4.

**Table 4:** Relative percentage of prescribing canine and feline gastrointestinal consultations distributed by pharmaceutical classes of systemic antimicrobials and grouped according to the EMA categorisation of antibiotics for use in animals for prudent and responsible use.

	Dogs (*n* = 5,384 prescribing EHRs)	Cats (*n* = 1,010 prescribing EHRs)
	% prescription (95% CI)^a^	% prescription (95% CI)a
Systemic antimicrobial		
Category A: Avoid	**–**	**–**
Category B: Restrict	1.90 (1.58–2.32)	20.10 (17.74–22.68)
Third generation Cephalosporin Fluoroquinolone	0.5 (0.3–0.7) 1.4 (0.4–2.4)	19.0 (15.4–22.5) 1.1 (0.3–2.0)
Category C: Caution	40.65 (39.27–42.02)	42.40 (39.36–45.45)
First generation Cephalosporin Second generation Cephalosporin Clavulanic acid potentiated amoxicillin Metronidazole and spiramycin (macrolide) Clindamycin Other Lincosamides Macrolide (others) Amphenicol	0.3 (0.2–0.5) 0.03 (0–0.08) 33.1 (29.9–36.2) 5.62 (3.3–8.0) 0.3 (0.2–0.5) 0.2 (0.0–0.3) 0.9 (0.0–1.9) 0.2 (0.1–0.4)	– – 37.1 (32.8–41.3) 3.4 (1.9–5.0) 0.8 (0–1.8) – 0.8 (0.0–2.1) 0.3 (0–0.6)
Category D: Prudence	56.05 (54.42–57.67)	36.59 (33.62–39.55)
Aminoglycoside Amoxicillin Tetracycline Other β-lactams^b^ Penicillin Metronidazole Fusidic acid Potentiated sulphonamide	1.5 (1.14–1.8) 15.73 (11.5–19.8) 1.0 (0.5–1.5) 0.1 (0–0.2) 0.02 (0–0.05) 33.9 (28.7–35.8) 3.0 (2.5–3.4) 0.8 (0.4–1.3)	0.5 (0.07–0.9) 27.7 (23.1–32.3) 0.5 (0.1–0.9) – – 5.4 (3.7–7.1) 2.4 (1.5–3.3) 0.09 (0.0–0.3)
Other antimicrobial agents^c^	1.40 (0.4–2.5)	1.0 (0.1–2.0)

^a^Percentage of total prescribing EHRs within the antimicrobial group, 95% confidence interval. ^b^Ampicillin and Cloxacillin. ^c^Multiple classes; Polymyxin b sulphate, mupirocin, novobiocin, thymol, and bronopol.

### 3.4 Factors associated with systemically administered AMP

Univariable results are presented in the Supplementary material (Supplementary Table S1 for canine GI consultations, and Supplementary Table S2 for feline GI consultations). The results of multivariable mixed effect logistic regression models for dogs and cats (Tables 5, 6, respectively), assessing the association between a number of factors related to animal signalment, questionnaire responses, and probability of systemic AMP, showed that in both species, presentations classified by the attending veterinary professional as moderate/severe were associated with significantly increased odds of systemically administered AMP when compared with mild GI presentations (canine moderate/severe odds ratio, OR, 1.86, 95% CI 1.65–2.07; feline moderate/severe OR 2.03, 95% CI 1.68–2.46). In addition, the presence of diarrhoea, both non-haemorrhagic and haemorrhagic, was associated with significantly increased odds of systemically administered AMP when compared with the absence of diarrhoea in GI presentations (canine non-haemorrhagic diarrhoea OR 2.11, 95% CI 1.91–23.33; feline non-haemorrhagic diarrhoea OR 1.77, 95% CI 1.48–2.11; canine haemorrhagic diarrhoea OR 4.22, 95% CI 3.80–4.68; feline haemorrhagic diarrhoea OR 3.05, 95% CI 2.44–3.82). GI presentations of between 2 weeks and 1-month duration and presentations of more than 1-month duration were associated with significantly decreased odds of systemically administered AMP in both canine and feline GI consultations compared with GI presentations of less than 2 days duration (canine >2 weeks and < 1-month duration OR 0.73, 95% CI 0.60–0.88; canine ≥1-month duration OR 0.61, 95% CI 0.51–0.72; feline >2 weeks and < 1-month duration OR 0.49, 95% CI 0.35–0.68; feline ≥1-month duration OR 0.39, 95% CI 0.30–0.50).

**Table 5:** Results from a finalised multivariable mixed effects logistic regression model, modelling on a case level the presence of systemically administered antimicrobials against a series of risk factors in canine GI consultations (*n* = 18,829 EHRs).

Random effect	Variance	Standard deviation	Variable	Category	β	SE^a^	OR^b^	Lower CI^c^	Upper CI	*P*
Practise Site	0.42 0.19	0.65 0.43		Intercept	−2.08	0.07	0.13	0.11	0.15	–
			Consultation episode	First visit Revisit	−0.15	0.05	1.00 0.99	– 0.89	– 1.09	– 0.76
			Severity	Mild Moderate/Severe	– 0.62	– 0.06	1.00 1.85	– 1.65	– 2.07	– <0.001
			Diarrhoea	Absent Non-haemorrhagic Haemorrhagic	– 0.75 1.44	– 0.05 0.05	1.00 2.11 4.22	– 1.91 3.80	– 2.33 4.68	– <0.001 <0.001
			Duration	≤ 2 days ≥ 3 days and ≤ 2 weeks > 2 weeks and < 1 month ≥ 1 month Do not know	– 0.14 −0.32 −0.50 −0.77	– 0.04 0.10 0.09 0.29	1.00 1.15 0.73 0.61 0.46	– 1.07 0.60 0.51 0.26	– 1.24 0.88 0.72 0.82	– <0.001 0.001 <0.001 0.008
			Continuous factor Age (years)	Age–linear	0.033	0.004	1.03	1.02	1.04	<0.001
			Interaction terms							
			Consultation episode: Severity	Revisit: Moderate/Severe	−0.50	0.097	0.61	0.50	0.73	<0.001
			β Coefficient estimate ^a^Standard error ^c^95% Confidence interval ^b^Odds ratio							

Explanation of interaction term: First visit, mild presentation, OR = 1. Revisit, mild presentation, OR = 0.99. Revisit, mild presentation, OR = 0.99. First visit, moderate/severe presentation, OR = 1.85. Revisit, moderate/severe presentation, OR = 0.92.

**Table 6:** Results from a finalised multivariable mixed effects logistic regression model, modelling on a case level the presence of systemically administered antimicrobials against a series of risk factors in feline GI consultations (*n* = 4,508 EHRs).

Random effect	Variance	Standard deviation	Variable	Category	β	SE ^a^	OR ^b^	Lower CI ^c^	Upper CI	*P*
Practise Site	0.65 0.23	0.80 0.48		Intercept	−1.69	0.11	0.18	0.15	0.23	-
			Severity	Mild Moderate/Severe	– 0.71	– 0.10	1.00 2.03	– 1.68	– 2.46	– <0.001
			Diarrhoea	Absent Non-haemorrhagic Haemorrhagic	– 0.57 1.12	– 0.09 0.11	1.00 1.77 3.05	– 1.48 2.44	– 2.11 3.82	– <0.001 <0.001
			Duration	≤ 2 days ≥ 3 days and ≤ 2 weeks > 2 weeks and < 1 month ≥ 1 month Do not know	– −0.12 −0.72 −0.95 −0.77	– 0.09 0.17 0.13 0.29	1.00 0.89 0.49 0.39 0.46	– 0.75 0.35 0.30 0.26	– 1.06 0.68 0.50 0.82	– 0.199 <0.001 <0.001 0.008
β Coefficient estimate ^a^Standard error ^b^Odds ratio ^c^95% Confidence interval										

**Figure 2:**
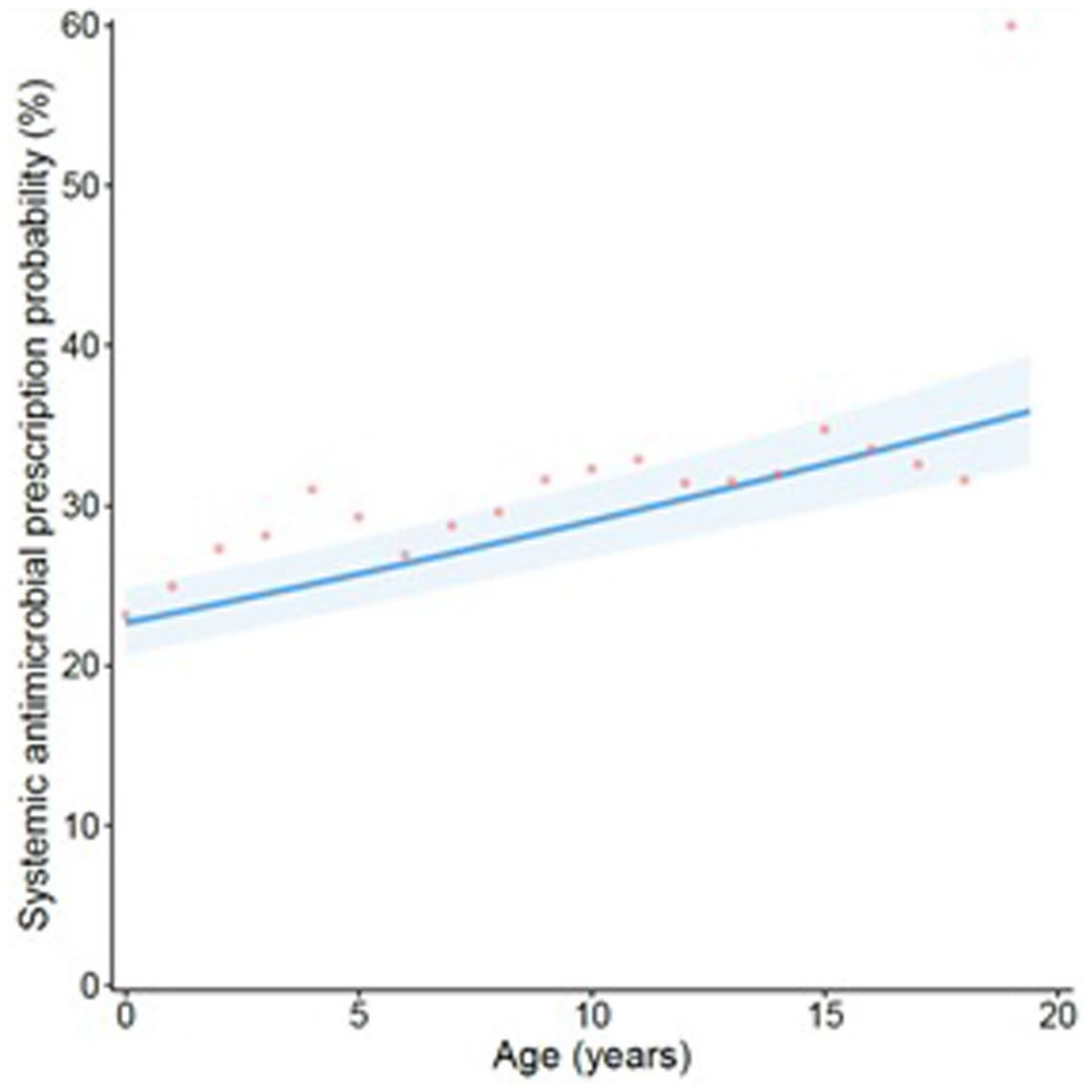
Projection of the probability of a systemically administered antimicrobial being prescribed in GI canine consultations when considered against age at consultation (in years). The line refers to predicted probability, with shading corresponding to a 95% confidence interval. Points are plotted to show original data points expressing the average percentage of consultations of each relevant age group (rounded to 0.5-year groups) in which a systemically administered antimicrobial was prescribed.

In canine GI consultations, inclusion of an interaction between consultation episode and severity provided the best fit. Moderate/severe presentations were associated with increased odds of systemically administered AMP in first-visit consultations when compared to other consultations (see footnote Table 5). In canine consultations, systemically administered AMP probability increased with age. A linear term provided the best fit for canine presentations (Figure 2).

### 3.5 Thematic analysis of the clinical narrative of HPCIA-prescribing consultations

**Figure 3:**
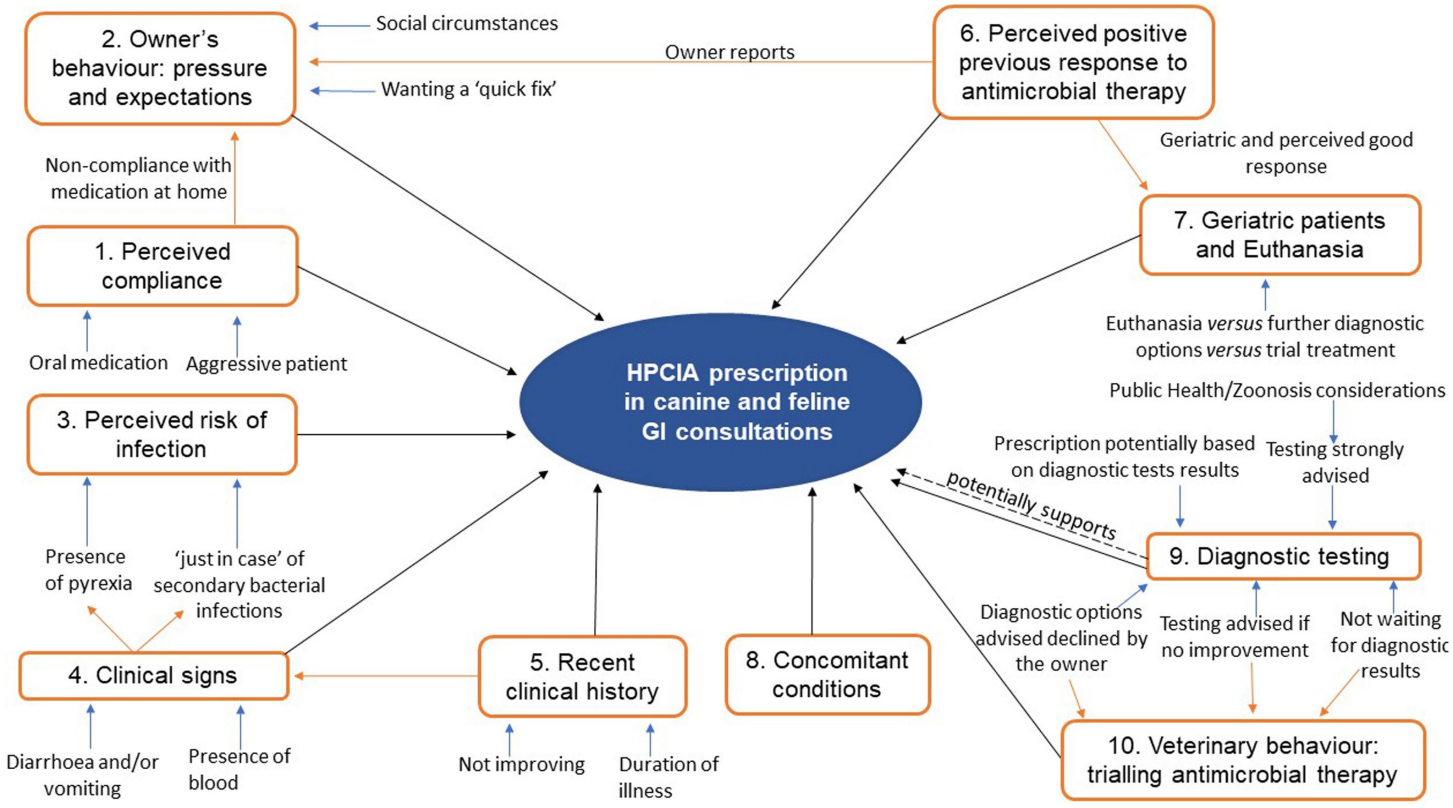
Thematic map demonstrating the relationship between several minor and 10 major themes around HPCIA prescription, resulting from the thematic analysis of the clinical narrative content of 516 canine and feline gastrointestinal (GI) consultations. Orange arrows highlight the relationships between major themes. Blue arrows highlight the connection of minor with major themes.

The 516 GI consultations where an HPCIA was prescribed included 334 from cats and 182 from dogs. Third-generation cephalosporins accounted for 21% (*n* = 38/182) of canine HPCIA-prescribing consultations, and they were the most frequently prescribed class of HPCIA in cats (93%, *n* = 310/334). Fluoroquinolones were prescribed in 4% (*n* = 15/334) of feline and 48% (*n* = 87/182) of canine HPCIA-prescribing consultations. Macrolides accounted for 3% (*n* = 9/334) of feline and 31% (*n* = 57/182) of canine HPCIA-prescribing consultations. Of these 516 clinical narratives, justification or reasoning-related content around HPCIA prescription was evident in 23% (*n* = 117/516) of consultations. There were 13 consultations that did not have any clinical narrative (blank free-text). Thematic analysis of the narratives where justification or reasoning-related content was found identified 10 major themes underpinning reasoning around HPCIA prescription. Figure 3 shows the thematic map and the relationship between several minor themes and 10 major themes.

Theme 1: Perceived compliance.

Perceived animal compliance was identified in the clinical narrative, commonly associated with expected owner difficulties with oral medication in felines and aggressive patients although non-compliance with oral medication was also identified in dogs.

*‘Dog not compliant for oral medication at home. Very little appetite and still passing mucoid diarrhoea.’* [dog].

*‘As < <name> > is hard to handle (caretaker will have problem with tablets) and there may be a problem to bring her in tomorrow I am givin [giving] her convenia.’* [cat].

Moreover, a perceived inability or unwillingness of the owner to administer oral antimicrobial therapy was identified as influencing the antimicrobial formulation selected and, therefore, the substance. In some cases, the veterinary professional explicitly stated changing their first-choice antimicrobial based on perceived compliance.

*‘Could not tablet, offered clavapet to be crushed but O [Owner] preferred to try convenia.’* [cat].

*‘O [Owner] would be unable to give penicillin course orally so convenia injected’* [dog].

Theme 2: Owner’s behaviour: Decision-maker pressure and expectations.

Recorded in the clinical narratives was a discussion about diagnostic testing and further investigation, where the owner declined this option, leading the practitioner to prescribe a medical treatment empirically, including antimicrobial therapy.

*‘Discussed further investigation (feaces [faeces] sample + bloods-FIV, FeLV + xray) vs treatment. O. [Owner] elected 2nd option by now and will see after Christmas.’* [cat].

Distinct reasons were identified in some clinical narratives, potentially shaping prescribing behaviour. These included pressure by the owner related to non-compliance with oral medication administration, and the inability or unwillingness to pay for further investigations.

*‘The owner has money problems and < <identifier> > is not insurred [insured]. I offer the option of giving AB [antibiotics] (no good with tablets so Convenia seems like only option) (…) The owner is aware that we are treating blind and that we may not be able to help her as much as we could because we do not know what the real problem is.’* [cat].

In some clinical narratives, there was evidence that pressure from the owner was shaping the veterinarian–client interaction. In these circumstances, the veterinary professional recorded a remark of yielding to the owner’s expectations in order to preserve agreement with the client even when the decision for prescribing is against their clinical judgement or when the veterinary professional would prefer other options to be implemented.

*‘adv [advised on] a number of ddx [differential diagnosis]. plan: adv [advice] bloods, o [owner] prefer to trial tx [treat] first as responded to ab previously. [I was] not keen to give ab but as has improved previously and o reluctant to investigate felt was best to cover’* [dog].

*‘O [Owner] going away on Monday so wants to get sorted. (…) offered bloods but O [Owner] would rather try treating first - if no better by tomorrow then recheck and would advise bloods + IVFT [intravenous fluid therapy] if dog has become dehydrated. Other dog had a bout of this recently and O [Owner] felt that she only improved with Abs [antibiotics] so wanted me to give convenia. Did explain that gastroenteritis is often viral but she still wanted Abs [antibiotics].’* [dog].

Theme 3: Perceived risk of infection.

The perceived risk of infection or risk of secondary infection, often associated with the presence of pyrexia, was noted in the narrative to influence prescribing decisions. However, some veterinary professionals expressed uncertainty about the aetiology of the pyrexia and/or underlying cause.

*‘(…) Has muild [mild] pyrexia so susp [suspected] infectious cause leading to oesophagitis or pharyngitis. Plan Abs [antibiotics]’* [cat].

*‘nad [nothing abnormal detected] on clinical exam. Suspect viral? can trial on antibiotics in case there’s any secondary’* infection?’ [cat].

*‘temperature 39, checked twice. Abdominal palpation limited. Gave convenia to cover possible infections..’* [cat].

In one clinical narrative, the veterinary professional justified AMP by the perceived risk of bacterial translocation in the gastrointestinal tract, implying a possible intestinal mucosal compromise and/or an infectious process involvement in the GI clinical presentation.

*‘convenia (to reduce risk of bacterial translocation)’* [cat].

Theme 4: Clinical signs.

The prescription of an HPCIA was also justified in association with other clinical signs that the animal presented with, including diarrhoea. It also seemed that the presence of blood in diarrhoea, or vomiting, was used to suggest a more severe disease warranting AMP.

*‘Diarrhoea 2 days, definitely some with blood this am [morning], no vom [vomit], still (…) As blood given abios [antibiotics]. T 38.4’* [cat].

‘*Convenia given as mild high temperature and blood in faeces.’* [cat].

*‘probind and convenia.to clear up diarrhoea.’* [cat].

Theme 5: Recent clinical history.

Factors related to the recent clinical history, such as the duration of the clinical signs, were also given as justification for AMP, as illustrated below.

*‘Given duration of signs I have decided to start Baytril.’* [cat].

The end of a previously prescribed antimicrobial therapy associated with recurrence of GI clinical signs also prompted AMP a second time regardless of further diagnostic testing advised by the veterinary professional. In some cases, diagnostic testing was initiated but AMP happened before results of the diagnostic test were made available.

*‘D+ [diarrhoea] recurred soon after end of AB’s [antibiotics] (…) initially prolonged ab [antibiotic] course if recurs despite this will require further investigation’* [dog].

*‘so advise f + [faecal] sample but treat in meantime.*’ [dog].

Theme 6: Perceived positive previous response to antimicrobial therapy.

Some veterinary professionals explicitly mentioned a perceived positive response to previous antimicrobial therapy. This positive response may also be used by the owner as an attempt to compel the veterinary professional to prescribe an antimicrobial agent, thus linking to owner pressure.

*‘History of D+ [diarrhoea] and good response to antibiotics all the way back to 2012.’* [cat].

*‘Has improved w/ [with] antibiotics, so seven days more’* [cat].

*‘o [owner] prefer to trial tx [treatment] first as responded to ab previously.’* [dog].

Theme 7: Geriatric patients and euthanasia.

Additionally, the geriatric condition of patients was linked with discussion around euthanasia. Frequently, concomitant conditions were mentioned, and antimicrobials were prescribed to reduce the perceived risk of infection in cases where the owner declined further investigation or whilst the owner considered euthanasia. Thus, in this context, AMP appeared to be an empirical treatment approach in either trying to postpone euthanasia or whilst waiting for a decision on the part of the owner.

*‘V+/D+ [vomiting and diarrhoea]. Often has episodes of gastroeneteritis [gastroenteritis] (…) O [Owner] takes her to a differemnt [different] vets where she usually responds to cernia [cerenia] and convenia. This time she has v + [vomited] 2–3 times and also has d + [diarrhoea]. No blood in d + [diarrhoea]. (…) Is an old girl and as has recurrent episodes that are ab [antibiotic] responsive given convenia as well today.’* [dog].

*‘Continuing problem with vomiting. (…) She is polydipsic but has been for years and has been investigated for this. (…) Ideally we would investigate eg BT, UA, scan/xray abdo [abdominal]* etc. *Unfortunately costs an issue so we have decided to postpone euth [euthanasia] and try meds.’* [cat].

*‘*Var*ious issues (…) had some d + [diarrhoea]. Lost 0.5 kg. (…) O [Owner] not sure whether time to euthanasia or no. Cat seems bright and comfortable. Discuss full workup, bloods, imaging wvt bladder surgery, O [Owner] not keen. Opt [Opted] for medical manage with antibiotics and nsaid [Non-steroidal anti-inflammatory] for now, O [Owner] aware palliative.’* [cat].

*‘Re/ex [Re-examine] – GI [Gastrointestinal] Issues. (…) Has lost further weight (40 g) and still d + [diarrhoea] which owner is noting in garden. (…) Recommended repeat abdo [abdominal] ultrasound today to reassess small bowel and look for fluid but owner declines. As had been vomiting quite severely prior to the last vitbee injection, advise convenia to reduce risk of e.coli reflux into b.duct [biliary duct], p.duct [pancreatic duct] and some concerns about feel of abdo [abdomen]. (…) O [Owner] considering put to sleep if not improving.’* [cat].

Theme 8: Concomitant conditions.

Concomitant conditions were commonly mentioned, which, combined with other factors, may influence AMP. In most of such narratives, it was difficult to establish which clinical signs predominantly led to AMP. Nonetheless, the existence of other clinical signs or concomitant conditions (e.g., dermatitis) was rarely associated with narrative content related to bacterial culture and antimicrobial susceptibility testing, suggesting that empirical AMP with advice for further investigation in case of no improvement or recurrence was common.

*“Liquid faeces for 2d [days]. (…) Clinical exam unremarkable except peri-anal dermatitis. (…) Antibs [antibiotics] given for both GIT [gastrointestinal tract] and skin - would have preferred metronid for GIT but cat will not take oral meds.”* [cat].

*‘sick thursday and friday last week and runny diarrhoea (…) o [owner] noticed blood in urine once last time (…) Has had urine crystals before so cannt [cannot] rule out but sounds like general infection Start antibiotics rx [re-examine] 5 days If urination worse phone us asap and try to get a urine sample.’* [dog].

Theme 9: Diagnostic testing.

Clinical narratives mentioning diagnostic testing, such as haematology, imaging (ultrasound), and bacterial culture, were infrequently identified. When diagnostic testing options were mentioned, they were often related to the near future, with HPCIA prescription happening in the current consultation. Thus, HPCIAs were prescribed often before any faecal culture results were available, including narratives suggesting that antimicrobial therapy might be adjusted after faecal culture results. In one narrative, the slow turnaround of results was mentioned, implying justification for starting antimicrobial therapy empirically. In addition, public health considerations regarding concerns about zoonotic agents were identified in two narratives.

*‘Presented as still d + [diarrhoea] watery and frequent and now v + [vomiting] again. (…) Poss inf [possible infection], recommend f + [faecal] sample but results are slow so start meds in meantime.’* [cat].

*‘Recheck colitis. Prelim [Preliminary] results isospora/Clostridia/Camp [Campylobacter]. (…) treatment as young children & immunosupressed [immunosuppressed] adult in household as a precaution. Adv [Advised] cannot say if pathogenic strain.’* [dog].

*‘DAIRRHOEA [DIARRHOEA], WELL IN SELF. (…) Pro-bind to help restore the flora. Asked for bringing a faecal sample (comprehensive Faecal) for testing again to see if Cryptosporidium still on. NOTE: O [Owner] is advanced pregnant and it’s a zoonotic disease. Warned O [Owner] about this.’* [cat].

Diagnostic results from imaging (ultrasound) and haematology were mentioned in a discussion around differential diagnosis; however, bacterial culture results were not mentioned, suggesting likely empirical AMP.

*‘still same and bloods indicate either infection/inflammation/neoplasia will send off for electriophoresis [electrophoresis] and treat in meantimne [meantime].’* [cat].

*“3 days with metronidazole, and concenia [convenia] due to v high wbc, [white blood cells count]*? *coccy cysts? incidental or immunocompromised? poss chk felv status’* [cat].

*‘Bloods suggestive of bacterial overgrowth or a shunt, therefore admitted for ultrasound investigation. Full scan (…) Pancreas normal. Intestinal content fluid, therefore suggests chronic malabsorption/bacterial overgrowth, therefore started treatment for this.’* [dog].

Theme 10: Trialling antimicrobial therapy.

One factor, apparently led by the veterinary professional, was to trial an empirical antimicrobial therapy as a first-line treatment. Veterinary professionals often recorded advising faecal sampling and/or further investigation if the introduced therapy was deemed to fail. This is in contrast to consultations where pressure to prescribe antimicrobials seemed to come from the owner despite the veterinary professional recommending other options (theme 2).

*This morning has been sick 5 times, bile content. (…) Treatment support for gastroenteritis. Inj [injection] of antbs [antibiotics], ranitidine and cerenia given. Disc [Discussed] if still sick or deteriorates for the next hours to bring her back for further investigation.’* [cat]’.

*‘v + [vomiting]. O [Owner] reports been v + [vomiting] after eating over past 24/48 h. (…) possible gastroenteritis? given convenia injection. (…) if does not settle or v + [vomiting] reoccurs phone and may take bloods.’* [cat].

## 4 Discussion

This mixed-methods study combined analysis of the content of EHRs with structured questionnaire responses to profile canine and feline GI presentations in United Kingdom veterinary primary care, along with their treatment, with a particular focus on AMP. Additionally, we investigated the reasoning and justification underpinning HPCIA prescription using thematic analysis of narratives from GI consultations, identifying 10 major themes. This approach provides new opportunities to understand antimicrobial prescribing and decision-making in consultations for GI disease and could be applied to other prescription events to unlock previously untapped data recorded within EHRs, thereby promoting antimicrobial stewardship at individual and population levels.

Consistent with previous studies (14), most cases of GI disease were considered mild in severity, commonly associated with non-haemorrhagic diarrhoea and non-haemorrhagic vomiting (8, 38, 39). Despite this, systemically administered AMP was common, occurring in 29% of canine GI consultations and in 22% of feline GI consultations (Table 3). Guidelines clearly state that feline and canine acute GI presentations, including dogs with haemorrhagic diarrhoea, that are systemically well do not require any antimicrobial therapy (16). This current figure was lower than previous studies where antimicrobials were used in 36–39% of canine and 26–29% of feline GI consultations (3, 39), consistent with a general reduction in AMP in veterinary practise (39). The most commonly prescribed systemically administered antimicrobial in feline consultations was clavulanic acid potentiated amoxicillin, which, according to the EMA “Categorisation of antibiotics for use in animals for prudent and responsible use,” should be used with caution (Category C) (15). In canine consultations, the most commonly systemically administered antimicrobial was metronidazole, which is again consistent with previous findings (3, 39). It has been described that the frequent use of metronidazole in canine GI presentations may be associated with a perceived anaerobic bacterial aetiology, such as *Clostridium perfringens*, despite its controversial role as an agent causing GI disease (12, 14). According to practise-level guidelines on AMP, metronidazole is recommended only for chronic diarrhoea/enteropathy treatment after all other diagnostic options and empirical therapy possibilities have been exhausted (14, 16). Therefore, the frequent use of metronidazole in canine GI presentations identified here suggests limited compliance with published guidelines.

We used a six-phase thematic analysis as defined by Braun and Clarke (31) to analyse what the veterinary professional recorded in EHRs at the time of the decision-making process around HPCIA prescription. Our analysis shows that most narratives (399 of 516) lacked any recorded detail for the decision-making process around HPCIA prescription; it is unclear if this is due to lack of consideration or just lack of recording. On one level, this is clearly a limitation of the chosen study methodology that can only describe what is recorded within the clinical narrative. Here, complementary social approaches, such as ethnographic observations, could help develop a deeper contextual understanding of what drives current behaviours and what needs to be targeted to enable change. Such an approach could also shed light on other factors, such as social norms established in the workplace, that were entirely absent in our analysis. That said, given the current importance of HPCIAs, we might expect more justification and/or reasoning to be recorded within the EHRs around the decision for prescribing such critical antimicrobial agents, especially considering that the majority of HPCIA systemically prescribed identified in our study were third-generation cephalosporins and fluoroquinolones, which are classed under the “Category B–Restrict” according to the EMA “categorisation of antibiotics for use in animals for prudent and responsible use” (15, 24).

Systemically administered HPCIA prescription was found in both canine and feline consultations. Whilst in dogs, HPCIAs were prescribed in less than 1% of consultations; in cat consultations, HPCIAs were prescribed in 5% of GI presentations, most frequently third-generation cephalosporins (Table 4). This is consistent with previous studies that identified the duration of action of this preparation (long-acting, 14 days) and its ease of administration (injectable) as factors underpinning its extensive use, particularly in those animals that are non-compliant with oral medication at home (22, 40). This was supported by our qualitative findings, where perceived compliance, whether non-compliance by the owner and/or the animal with oral medication at home, or with aggressive and/or difficult-to-handle patients, was recorded to justify HPCIA prescriptions in cats and to a lesser extent dogs (theme 1). In a previous qualitative study that utilised semi-structured interviews, veterinary participants were conflicted about whether the use of this pharmaceutical product was likely to increase or decrease AMR (23). In the absence of other authorised long-acting formulations, it seems likely that compliance may still be influencing prescribing behaviour towards third-generation cephalosporins (3). Third-generation cephalosporins are considered ‘highest priority critically important antimicrobials’ for human medicine by the World Health Organisation (24). Their usage can contribute to selective pressure in bacterial populations, hence increasing the risk of carriage of resistant bacteria (22, 24, 41, 42). For these reasons, and although a veterinary formulation of third-generation cephalosporin (cefovecin) is authorised for use in small animals in the United Kingdom, its use should be carefully considered and according to EMA. “These restricted antibiotics should only be used for the treatment of clinical conditions when there are no alternative antibiotics in a lower category that could be clinically effective and use should be based on the results of antibiotic susceptibility testing, whenever possible” (15). Hence, studies similar to ours that identify the ongoing use of third-generation cephalosporins in feline patients are particularly worrying. Identifying common prescription drivers allows us to promote the reduction of these and other antimicrobial classes considered critically important and will be vital for effective antimicrobial stewardship (43).

Consistent with previous studies, the use of diagnostic tests was infrequently recorded in both canine and feline consultations, with faecal bacteriology/parasitology being recorded in less than 8% of consultations in both populations (25, 38, 39). In feline consultations, the most commonly recorded diagnostic option was haematology/biochemistry (12% of feline consultations). The frequently mild nature of the reported disease, the majority of which being first-visit consultations, may underlie the infrequent use of diagnostic testing options in both species and suggests that many, if not most AMPs described in these GI consultations, were empirical. The overall lack of recorded evidence for bacterial culture and antimicrobial susceptibility testing, particularly prior to HPCIA use, suggests a fundamental departure from international guidance relative to the scientific advice on the categorisation of antimicrobials for use in animals for prudent and responsible use (15). These guidelines state that the use of antimicrobials that are critically important in human medicine should be restricted (Category B) to mitigate the risk to public health. These antimicrobials should only be considered when there are no other antimicrobial agents from the categories below (Categories C and D) that could be clinically effective, and their use should be based on antimicrobial susceptibility testing, wherever possible (15). Whilst we cannot preclude unrecorded testing taking place in some consultations, this represents a key area for practise education. The decision-making process around AMP can encompass a multitude of complex factors (22). Indeed, a veterinary professional, when presented with a patient suffering non-specific GI signs, may suspect an ongoing disease process in another organ or system, which, with limited use of diagnostic testing, might not be promptly identified, subsequently leading to empirical AMP. Such AMP seemed to occur frequently without any recorded evidence of this being based on pressure from the owner, suggesting that sometimes antimicrobial therapy was a primary clinical approach to a case presenting with such GI signs.

Multivariable modelling showed that the presence of non-haemorrhagic diarrhoea was significantly associated with increased odds of systemic AMP for both dogs and cats. This is consistent with previous studies where the presence of diarrhoea increased the probability of AMP to companion animals (3, 8, 44) and likely reflects a perceived bacterial aetiology and associated concerns of missing an infection and subsequent clinical deterioration in the absence of antimicrobial therapy. This is supported by our thematic analysis as the perceived risk of infection often associated with the presence of pyrexia and/or other clinical signs was used to justify prescribing HPCIAs in GI consultations (themes 3 and 4). Mitigation of the perceived risk of infection and the behaviour of *‘cover with antimicrobials just in case’* were captured in several clinical narratives (theme 3). This behaviour was previously described as a key driver of inappropriate prescribing related to feelings of fear of missing an infection that could negatively impact either the animal or the reputation of the practitioner (34). Other clinical signs, including haematochezia, haematemesis, and the presence of diarrhoea itself, were identified in the clinical narrative as clinical signs justifying the prescription of HPCIAs (theme 4). In one case, a perceived risk of bacterial translocation across the GI tract was associated with AMP. Haemorrhagic gastroenteritis might reflect a breach of intestinal integrity (45) with an associated risk of bacterial translocation, septicaemia, and potentially fatal septic shock (46). However, whilst bacterial translocation in veterinary patients has been documented, its role in critical illness is yet to be established (47). Further studies are warranted to document the frequency of bacterial translocation and whether these patients are at an increased risk for septic complications (47). Moreover, a recent study showed no difference in the incidence of bacterial translocation and no improvement in disease severity indices, laboratory parameters, length of hospitalisation, or mortality rates between canine patients with acute haemorrhagic diarrhoea receiving amoxicillin-clavulanic acid versus placebo (45, 48). Antimicrobial administration has also been shown to induce translocation from native commensal bacteria and promote an inflammatory response (47, 49). Establishing a consistent definition of sepsis risk in primary care, including potential new diagnostic markers would inform future effective antimicrobial stewardship (14).

In addition, when an interaction between episodes of consultation and severity was considered, the probability of systemically administered AMP in canine GI consultations was higher for moderate/severe presentations in first visit consultations. This likely reflects the attempt to address clinical concerns around the involvement of infectious agents when an animal is presented for the first time with a moderate/severe GI presentation.

In the present study, the owner’s behaviour, manifested by recorded evidence of pressure and expectations, was also identified as an important factor around prescription (theme 2). Examples included wanting a *‘quick fix’* of the animal’s condition, declining diagnostic options, social circumstances such as going on holidays, or difficulty affording further clinical investigations. Of course, these may not be drivers specific to HPCIAs, and further analysis is warranted to assess if similar themes are associated with non-HPCIA-prescribing consultations. Veterinary practitioners from a previous qualitative study affirmed feeling pressure from owners to prescribe antimicrobials, although most attested that they did not yield to such expectation (22). Nonetheless, and consistent with our findings, it was also shown that if the owner expressed inability or unwillingness to pay, veterinary practitioners affirmed they would consider changing their first choice of substance (22). In addition, owners reporting a previous positive experience (theme 6) with an HPCIA was perceived as pressure by the veterinarian to prescribe the same antimicrobial agent. Owner pressure is complex and multifaceted and may lead to behaviour that in some circumstances goes against the veterinarian’s intent of action based on their beliefs, scientific knowledge, training, or current guidelines (22, 23, 34). Such behaviour was evident in the clinical narrative. Attending veterinarians sometimes reflected that the HPCIA prescription was either empirical (e.g., *‘felt was best to cover’*) or that pressure from the owner for a certain therapy had eventually influenced their prescribing behaviour (e.g., *‘O felt that she only improved with Abs so wanted me to give convenia’*). To address the particular challenges identified in this study, implementation of strategies such as the ‘non-prescription form’ (50) developed by BSAVA/SAMSoc, could help to address client anxiety over AMP.

In dogs, we found the probability of systemically administered AMP consistently increased with age. This supports a previous qualitative study based on semi-structured interviews where treatment of elderly animals (e.g., one with a compromised immune system), or where owners were ‘particularly worried’, was associated with AMP for therapy of non-infectious gastritis (22). Due to the nature of the study itself, which is based on the clinical narrative produced by the veterinary professional at the moment of the consultation, our study was unable to extensively take into account concomitant conditions, which may be present in cases in elderly animals, and could, therefore, influence the decision-making process around AMP in such cases. However, in the future, access to EHRs should allow the impact of comorbidities on prescription to be further evaluated.

Consistent with previous studies, GI nutraceuticals were frequently dispensed, particularly to dogs (13, 14). Indeed, a recent longitudinal study in dogs suggested nutraceutical use was rising and that a combination of dietary modification and gastroenteric nutraceuticals without the prescription of pharmaceutical agents including antimicrobials was associated with slightly improved odds of resolution of diarrhoeic clinical signs (14). Despite recent studies advocating that probiotics might be useful in aiding the resolution of infectious, non-infectious, or idiopathic diarrhoea in dogs, further study is warranted in companion animals to better understand the complex interaction between probiotics and their host environment, mechanism of action, and potential clinical impact (51, 52).

In this study, EHRs that had an associated questionnaire completed by veterinary professionals were included to allow for the characterisation of GI clinical presentations in dogs and cats. These mandatory structured questionnaires are automatic and randomly assigned to veterinary consultations classified by the attending veterinary professional as GI, using the MPC function of the SAVSNET window. This strategy aims to overcome issues related to the lack and/or variability of recorded details on the clinical narrative of EHRs, as previously described (14). This complementary questionnaire data allowed for further characterisation of veterinary-assessed parameters, such as case severity, and appraisal of parameters such as veterinary advice provided, or the diagnostic options used consistently. However, the use of questionnaire answers presents its own limitations, particularly related to possible response bias, which we hope to have mitigated with the robust size of this study. Future studies would allow for the development of novel text mining strategies to better identify and follow cases, un-tapping a greater number of cases within the SAVSNET database (14, 53).

The prescribing cascade establishes the requirement for the veterinary practitioner to prescribe and use authorised veterinary medicines when available (54, 55). Although this general legislative provision is necessary, this can also create a perverse pressure to prescribe a product that is licenced but not consistent with the principles of responsible antimicrobial use, to the detriment of products that, although un-licenced, are more aligned with responsible use. This is particularly relevant when considering long-acting characteristics and the ease of administration of licenced veterinary formulations that have an HPCIA as an active substance. Arguably, for promoting effective antimicrobial stewardship in the small animal veterinary sector, all stakeholders, including licensing authorities, the pharmaceutical industry, and veterinary practitioners, would need to work together to find strategies tailored to this specific part of the veterinary sector. This would potentially promote discussions where the principles of animal welfare are prioritised and safeguarded, as well as the need to have product formulations that meet the needs of day-to-day practise whilst developing and promoting marketed pharmaceutical products consistent with concepts such as antibiotic susceptibility bonus (56). This scheme relies on the effectiveness of the antimicrobial drug for treating target pathogens in the years subsequent to market entry. Importantly, widespread professional training (e.g., workshops, seminars, and webinars) is needed to ensure that currently published guidelines are widely disseminated, and that the veterinary professional has the necessary resources for an evidence-based decision around AMP, not only in canine and feline GI presentations, but also in other clinical presentations. The findings presented here can be used to inform the development of such training, ensuring it addresses the challenges identified here faced by practitioners. In addition, our observations can be incorporated into targeted interventions aimed at small animal veterinary practises to safely reduce unnecessary prescription of antimicrobials in GI presentations, particularly HPCIAs. Future initiatives using population health data and a data intelligence background may be valuable to provide targeted awareness messages around antimicrobial use to veterinary professionals in real time, particularly if it allows veterinary professionals to reflect on their own prescribing behaviour in real time. For example, a targeted awareness message when prescribing an HPCIA without mentioning antimicrobial susceptibility testing results may also empower veterinary professionals to initiate a discussion with the owner about responsible antimicrobial use.

In conclusion, the present study highlights, on the one hand, the value of quantitative approaches to better understand AMP practises in GI presentations, particularly to profile commonalities of canine and feline GI presentations, to characterise management strategies, and to investigate factors associated with systemically administered AMP. However, on the other hand, research to date has largely neglected the use of the clinical narrative as a source of information. Hence, we advocate the continued integration of more qualitative approaches to increase the potential of the unstructured text-derived data found in EHRs. Using a mixed-methods approach provided novel insights into the information recorded in EHRs by veterinary practitioners, recorded as is, at, or close to, the time of the clinical decision to prescribe in the consulting room, thus reflecting the factors deemed most important to the decision-making process. Such a holistic view of EHRs provides complementary evidence and insights into the veterinary decision-making process underpinning AMP in companion animals. This can be used to inform evidence-based policymaking, development of targeted health messages, and professional development, contributing towards effective antimicrobial stewardship. We advocate greater recording of justification for HPCIA prescription in EHRs; at a minimum, this would serve to promote clinical reflection. Finally, further interdisciplinary efforts are needed to ensure clinical compliance with currently published prescription guidance.

## Data availability statement

The data analyzed in this study is subject to the following licences/restrictions: the Small Animal Veterinary Surveillance Network (SAVSNET) collects electronic health records from participating volunteer veterinary practises and laboratories. The clinical data collected by SAVSNET is protected and regulated according the General Data Protection Regulation (GDPR) and Ethics Committee review of the University of Liverpool. The de-identified raw data supporting the conclusions of this manuscript can be made available by the authors, without undue reservation, to any qualified researcher. Requests to access these datasets should be directed to AR, savsnet@liverpool.ac.uk.

## Author contributions

AR, DS, and GP conceived the project idea and revised the manuscript. IF devised the implementation of the study, supervised by AR, DS, and GP. IF and GP analysed data and drafted the manuscript. FS-V is a co-investigator on the grant funding of this study and assisted with revising the manuscript. All authors contributed to the article and approved the submitted version.
