# Supplementary material for: A mixed-methods approach utilising electronic health records to examine antimicrobial prescription surrounding gastrointestinal clinical presentations in dogs and cats
Source: Front Vet Sci. 2023 Dec 12;10:1166114. doi: 10.3389/fvets.2023.1166114 (PMC10749927; doi:10.3389/fvets.2023.1166114)
Supplement: Supplementary file 1 [file Data_Sheet_1.pdf]

Supplementary Material

1 Supplementary Data

Results from univariable mixed effects logistic regression models.

Supplementary Table 1. Systemic antimicrobials in canine consultations: Parameter estimates from a series of univariable mixed effects logistic regression models, modelling on a case-level the outcome variable ‘presence of systemic antimicrobial prescription’ against a number of categorical and continuous risk factors.

Random effect	Variance	Standard Deviation	Variable	Category	β	SE [a]	OR [b]	Lower CI [c]	Upper CI	*P*
**Practice**	0.39	0.63	**Insurance status**	Uninsured (Intercept)	-0.96	0.06	-	-	-	-
**Site**	0.16	0.40		Insured	-0.02	0.04	0.98	0.90	1.06	0.59
			**Vaccination**	Unvaccinated (Intercept)	-0.94	0.06	-	-	-	-
				Vaccinated	-0.04	0.04	0.96	0.89	1.04	0.33
			**Neutered status**	Un-neutered (Intercept)	-0.98	0.06	-	-	-	-
				Neutered	0.02	0.04	1.02	0.95	1.09	0.64
			**Consultation episode**	First visit (Intercept)	-0.94	0.05	-	-	-	-
				Revisit	-0.11	0.04	0.90	0.83	0.97	0.006
			**Severity**	Mild (Intercept)	-1.05	0.05	-	-	-	-
				Moderate/Severe	0.44	0.04	1.55	1.42	1.69	<0.001
			**Faecal bacteriology/parasitology**	Absent (Intercept)	-0.97	0.05	-	-	-	-
				Present	0.02	0.07	1.03	0.90	1.16	0.71
			**Diarrhoea**	Absent (Intercept)	-1.78	0.07	-	-	-	-
				Non-haemorrhagic	0.72	0.05	2.05	1.86	2.26	<0.001
				Haemorrhagic	1.43	0.05	4.17	3.77	4.62	<0.001
			**Vomiting**	Absent (Intercept)	-0.79	0.06	-	-	-	-
				Non-haemorrhagic	-0.42	0.04	0.66	0.61	0.70	<0.001

	Haemorrhagic	-0.31	0.09	0.73	0.61	0.88	0.001
**Duration**	≤ 2 days (Intercept)	-1.01	0.06	-	-	-	-
	≥ 3 days and ≤ 2 weeks	0.22	0.04	1.24	1.16	1.33	<0.001
	> 2 weeks and < 1 month	-0.31	0.09	0.74	0.61	0.88	0.001
	≥ 1 month	-0.50	0.08	0.61	0.52	0.71	<0.001
	Do not know	-0.70	0.28	0.50	0.29	0.86	0.013
Continuous risk factor							
**Age (years)**	Intercept	-0.97	0.05	-	-	-	-
	Age – linear	0.14	0.02	1.15	1.12	1.19	<0.001

[a] *Standard error*

[b] *Odds ratio*

[c] *95% Confidence interval*

Supplementary Table 2. Systemic antimicrobials in feline consultations: Parameter estimates from a series of univariable mixed effects logistic regression models, modelling on a case-level the outcome variable ‘presence of systemic antimicrobial prescription’ against a number of categorical and continuous risk factors.

Random effect	Variance	Standard Deviation	Variable	Category	β	SE [a]	OR [b]	Lower CI [c]	Upper CI	*P*
**Practice**	0.58	0.76	**Insurance status**	Uninsured (Intercept)	-1.32	0.08	-	-	-	-
**Site**	0.21	0.46		Insured	-0.07	0.11	0.93	0.76	1.15	0.51
			**Vaccination**	Unvaccinated (Intercept)	-1.29	0.09	-	-	-	-
				Vaccinated	-0.08	0.08	0.92	0.79	1.08	0.31
			**Neutered status**	Un-neutered (Intercept)	-1.21	0.11	-	-	-	-
				Neutered	-0.16	0.10	0.86	0.71	1.04	0.11
			**Consultation episode**	First visit (Intercept)	-1.25	0.08	-	-	-	-
				Revisit	-0.26	0.08	0.77	0.65	0.91	0.002
			**Severity**	Mild (Intercept)	-1.46	0.08	-	-	-	-
				Moderate/Severe	0.62	0.09	1.86	1.55	2.23	<0.001
			**Faecal bacteriology/parasitology**	Absent (Intercept)	-1.32	0.08	-	-	-	-
				Present	-0.19	0.15	0.83	0.61	1.11	0.21
			**Diarrhoea**	Absent (Intercept)	-1.73	0.10	-	-	-	-
				Non-haemorrhagic	0.47	0.09	1.61	1.35	1.91	<0.001
				Haemorrhagic	1.04	0.11	2.83	2.27	3.53	<0.001
			**Vomiting**	Absent (Intercept)	-1.12	0.09	-	-	-	-
				Non-haemorrhagic	-0.47	0.08	0.63	0.54	0.73	<0.001
				Haemorrhagic	0.01	0.19	1.01	0.69	1.48	0.95
			**Duration**	≤ 2 days (Intercept)	-1.16	0.09	-	-	-	-
				≥ 3 days and ≤ 2 weeks	-0.02	0.09	0.98	0.83	1.16	0.84
				> 2 weeks and < 1 month	-0.66	0.17	0.52	0.37	0.72	<0.001

		≥ 1 month	-0.77	0.12	0.46	0.37	0.58	<0.001
		Do not know	-1.21	0.55	0.30	0.10	0.88	0.03
	Continuous risk factor							
	**Age (years)**	Intercept	-1.45	0.09	-	-	-	-
		Age - linear	-0.11	0.04	0.90	0.83	0.97	0.006
		Age - quadratic	0.12	0.04	1.12	1.03	1.22	0.008

[a] *Standard error*

[b] *Odds ratio*

[c] *95% Confidence interval*

2 Supplementary Data

List of abbreviations and clinical acronyms present in the analysed clinical narratives:

a/b – antibiotics

Ab('s) – antibiotics

Abdo – abdomen

Abios – antibiotics

Abx – antibiotics

Adv – advised

Am – morning

Antibs – antibiotics

Asap – as soon as possible

B. duct – biliary duct

Bact – bacterial

BG – Blood Glucose

BT – Blood Test

Camp – *Campylobacter spp.*

Campy – *Campylobacter spp.*

Chk – check(ed)

D+(++) – Diarrhoea

Ddx – differential diagnosis

dexf – dexamethasone

Disc – discussed

E.coli – *Escherichia coli*

Euth - euthanasia

F/S – faecal sample

f+ – faeces/faecal

FeLV – Feline Leukaemia Virus

FIV – Feline Immunodeficiency Virus

FPLi – Feline Pancreatic Lipase Immunoreactivity

GE – gastroenteritis

GI – gastrointestinal

Hols – holidays

IBD – Inflammatory Bowel Disease

Inf – infection

Infxn – infection

Inj – injection

IVFT – intravenous fluid therapy

Meds – medication/medical treatment

Metronid – metronidazole

mmol – millimoles

Mtz – metronidazole

NAD – Nothing Abnormal Detected

NSAID – non-steroidal anti-inflammatory drug

NSF – No Significant Findings

O – Owner

Opt – opted

P – plan

P. duct – pancreatic duct

Poss – possible

Re – re-examination

Re/ex – re-examination

re-ex – re-examination

RV – revisit

Rx – prescribed medication/re-examine (depending on the context)

Susp – suspect(ed)

T – temperature

Temp – temperature

TLI - Trypsin-Like Immunoreactivity

Tx – treatment

UA – Urinalysis

V+ – vomiting

Vom – vomiting

W/ – with

W/e – weekend

WBC – White Blood Cell
